# The Unexpected Hand Patient

**Published:** 2017-05-05

**Authors:** Andrew M. Swiergosz, Morton L. Kasdan, Bradon J. Wilhelmi

**Affiliations:** University of Louisville School of Medicine, Louisville, Ky

**Keywords:** malingering, reflex sympathetic dystrophy (RSD), complex regional pain syndrome (CRPS)

## Abstract

**Objective:** Physicians should be aware of patients trying to obtain a diagnosis for secondary gain. Malingering is a diagnosis that should be suspected when objective findings do not support the subjective symptoms and there is secondary gain. **Methods:** A series of 21 cases are presented that support this position. The charts of 21 patients with a diagnosis of reflex sympathetic dystrophy (chronic regional pain syndrome) and nonanatomic findings were evaluated. **Results:** The patients in this series were found to be malingering based on discrepancies between subjective symptoms and objective findings. **Conclusions:** The diagnosis of malingering should be based on thorough history, physical examination, electrodiagnostic studies, imaging studies, and evaluation of all medical records.

Individuals have been feigning symptoms of illness to obtain secondary gain for thousands of years. The illusion of an illness to achieve “a consciously desired end” dates back to biblical times. In Samuel I, while David is fleeing from King Saul, he avoided execution by King Achish of Gath through the feigning of a sickness. This instance is described in the following passage.

These words worried David and he became very much afraid of King Achish of Gath. So he concealed his good sense from them; he feigned madness for their benefit. He scratched marks on the doors of the gate and let his saliva run down his beard. And Achish said to his courtiers, “You see the man is raving; why bring him to me? Do I lack madmen that you have brought this fellow to rave for me? Should this fellow enter my house?”^1^

Malingering is described as the willful and deliberate feigning or exaggeration of illness.^2^ It was originally described as a means of avoiding military service.^2^ The word “malingerer” was introduced in 1785, in a publication titled “Grove's Dictionary of the Vulgar Tongue.” The term “malingerer” is said to be derived from the French *malingre*, meaning sickly or feeble, and was originally used in a military setting to describe persons who pretended to be sick so as to evade military duty.^2^ Malingering is defined by the *DSM-5* as “the intentional production of false or grossly exaggerated physical or psychological symptoms.”^3^ It is motivated by external incentives such as avoiding military duty, work, obtaining financial compensation, evading criminal prosecution, or obtaining drugs.^3^^,^^4^ Identifying the reason for secondary gain associated with malingering is crucial in the confirmation of a diagnosis. Typically, the secondary gain is apparent and directly related to the reason for presenting to the physician.^5^ This is especially relevant when patients are being evaluated for a disability with the possibility of financial compensation. One study found the incidence of malingering in litigation or compensation-seeking cases related to pain or somatoform disorders to be 33.5%.^6^ This is significant because there are more than 38 million Americans classified as disabled, more than 19 million of which are in their working years, from 18 to 64 years.^7^ The diagnosis of malingering can be difficult. Many times it is a diagnosis of exclusion. The confirmation of a diagnosis of malingering can be either observed or inferred. An example of an observed confirmation would be covert surveillance, such as a patient being videotaped in his or her natural environment doing something that would not be possible if the symptoms claimed were present. An inferred diagnosis is achieved by data acquisition and collection to see if what the patient is claiming can be proven by objective finding. An example of this would be a depressed patient who complains of poor appetite and sleep yet may be discreetly observed to always finish his meal, have the desserts, sleep soundly, interact appropriately with others, and not lose weight.^8^

We present a case series in which one of the authors (M.L.K.) evaluated 21 patients from 1979 to 1995 in which the patients were found to be malingering in legal cases where they were applying for compensation. Fourteen of the cases were workers’ compensation claims and 7 involved tort litigation. The patients arrived for evaluation with a diagnosis of reflex sympathetic dystrophy (RSD), now called chronic regional pain syndrome, and were asking for monetary compensation.

## SELECTED CASES

### Case 1

**Figure 1. Case 1:**
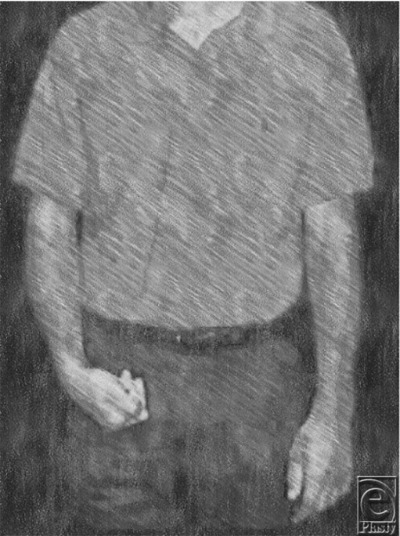
The nonanatomic position present at the time of the patient's evaluation.

This was a 23-year-old man with a history of blunt trauma to his right hand. While working, an electric motor rolled on his right hand and forearm, resulting in swelling and abrasions on the dorsum of his hand and elbow. There were no fractures seen on radiographs. No surgery or sutures were needed. He complained of constant pain for 15 months. He was diagnosed with RSD without objective imaging conformation. He received a cervical epidural block 3 months after the injury, a stellate ganglion block 4 months after the injury, and an intravenous block 5 months after the injury. He stated that none of these treatments helped his pain. Seven months after the injury, a dorsal column stimulator was implanted, which he stated relieved 50% of the pain. The patient presented 15 months after the injury for a medical evaluation as part of workers’ compensation litigation process. He claimed constant pain and the inability to use his right hand and arm. Upon physical examination, the patient held his right hand in a clenched fist posture with his fingers overlapping. He held his elbow and wrist in a fixed flexed posture and adducted against his body, as shown in Figure 1.

He would only abduct his arm minimally at the shoulder. It was noted that the patient was suntanned, including the axillary area on the right side and on his right chest. All finger nails appeared to be chewed short on both hands. He stated that he did not bite his nails on his right hand because he could not get his hand to his mouth. He claimed that he picked the nails of his right hand off with his left hand. He later stated that his right hand was too tender to be touched, yet there was no allodynia on examination. There was no obvious atrophy upon examination of the upper extremities. Mineralization of the bones was symmetrical on bilateral radiographs. The patient initially claimed that his right hand had been in this position for 6 months, while his wife later said he had held his hand in this position for 1 year. His wife stated that his hand did open while sleeping.

A private investigator was hired by the patient's former employer. The video surveillance showed the patient grasping and casting repeatedly with a fishing rod and reel in the hand that he claimed to be nonfunctional with a full range of motion.

### Case 2

This was a 35-year-old man who sustained a glass laceration of the volar left index finger at work. Examination in the emergency department by a hand surgeon revealed a 3-cm laceration involving a digital nerve and 2 flexor tendons. The nerve and tendons were repaired. He continued to have pain and was seen by multiple physicians. He was given the diagnosis of RSD without imaging conformation.

**Figure 2. Case 2:**
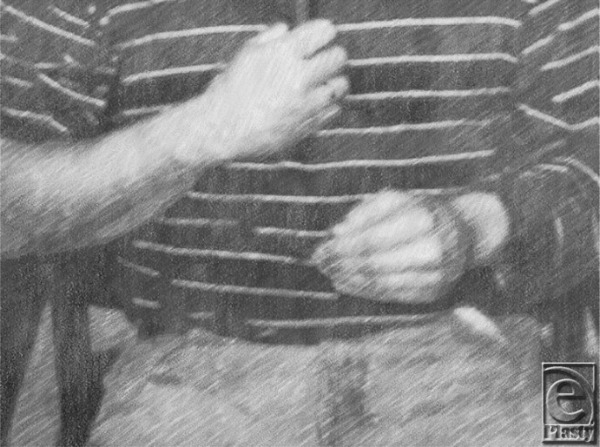
The fixed posture of the digits of the left hand.

The patient presented 30 months after the injury for an impairment evaluation for a workers’ compensation claim. He stated that he attempted to go back to work after the initial surgery while he was still in a cast. He says he was told that he was not wanted back at work until he could use both hands and without taking any pain medication. He described swelling and a burning pain in his left hand. He claimed a loss of extremity hair and said he could not tolerate clothing on the left hand. He would not attempt to move his digits. He held his arm flexed at the elbow and adducted against his body and the fingers in a fixed position as documented in Figure 2.

Electrodiagnostic studies revealed the left upper extremity to be within normal limits. There were no objective trophic changes, and diagnostic imaging found symmetric mineralization.

### Case 3

**Figure 3. Case 3:**
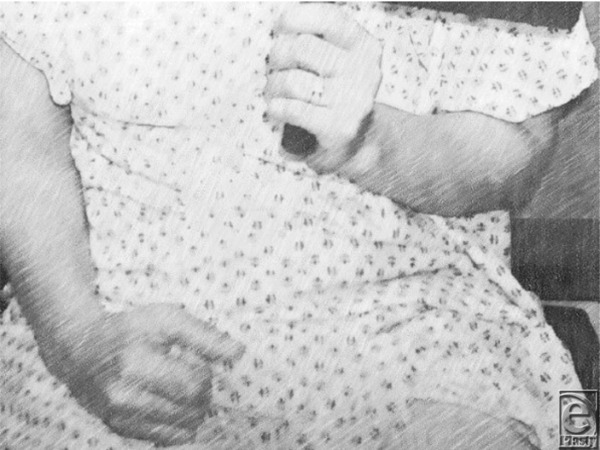
The patient claimed that her right hand could not be moved from the position shown in the artwork.

This was a 30-year-old woman who sustained a crush injury to her right hand at work. She was injured while repairing a machine that was started while she was still working on it. She was evaluated in an emergency department. No injury was documented. The patient presented 27 months after the injury for an evaluation for workers’ compensation claim. She denied prior injuries to her hands. The old medical records document 9 prior injuries. The patient had been diagnosed with RSD. She complained of her fingers being “stuck” in a clenched position and of severe pain in the entire right upper extremity. During examination, she held her right hand in a clenched position. The physical examination was normal except for the position of her hand as documented in Figure 3.

Her hand posture is not seen in patients with RSD. Patients with RSD will not hold their hands clenched, as it is too painful for them. The hair growth was symmetrical on the dorsum of her hands. There was no evidence of bone demineralization or osteoporosis in either hand upon imaging studies.

## DISCUSSION

The diagnosis of malingering is an important one to make in the clinical setting for several reasons. There is a significant cost to society due to malingering. Chafetz and Underhill^9^ estimate that malingering in Social Security Disability examinations occurs in 45.8% to 59.7% of adult cases. They go on to estimate that in 2011, the cost of malingering in medicolegal cases involving external incentive was $20.02 billion for adult mental disorder claimants alone.^9^ This also becomes important when considering the cascading effect on the economy. The cost of workers’ compensation insurance is passed on to the consumer, further driving up the price of consumer goods.^10^

It is important to rule out medical conditions that are causes of fixed postures versus malingering. There are conditions that present similar to these cases in this series that should be ruled out. The clenched fist syndrome is a condition in which the hand is held in a tightly clenched fist after minor trauma or surgery.^11^ Patients present with flexion contractures of the fingers without an anatomic lesion.^12^^,^^13^ This condition can be treated with interventions such as physical therapy, stretching, psychotherapy, and splinting.^12^^,^^13^ These patients may have a psychological illness and not be seeking monetary gain. This reinforces the importance of identifying malingering versus true psychopathology and neurological pathology such as stroke, dystonia, and true RSD so that appropriate treatment can be initiated.

Reflex sympathetic dystrophy is a complex of symptoms that is thought to be due to an interaction between central and peripheral processes.^14^ This occurs days or weeks after a trauma, and it is most prevalent in the upper extremity.^14^ Reflex sympathetic dystrophy may present with any assortment of the following symptoms: hyperalgesia, edema, stiffness, skin discoloration, trophic changes, hyperhidrosis, and stiffness.^15^ Unfortunately, litigation and compensation issues tend to propagate both complaints and medical management, as the psychosocial scenario may become contaminated with a secondary gain agenda from the patient.^16^ Patients with dysfunctional postures can be misdiagnosed as having RSD,^17^ as in this case series. The literature has documented that malingering may be misdiagnosed as RSD.^18^^,^^19^ When misdiagnosed with RSD, the patient may undergo the avoidable process of *medicalization*, which attempts to correct wrongly diagnosed physical complaints with invasive procedures.^20^^,^^21^ Medicalization can trigger the *nocebo effect* in somatizing patients,^22^^,^^23^ which represents negative prospects about symptoms that discourage the patient from taking an active role in recovery.^22^^,^^23^ In addition to undergoing unnecessary procedures, drug dependence can occur in these patients, as they are prescribed narcotics when their physicians give up after the failure of all attempted therapies aimed at the assumed cause of their continuous peripheral symptoms.^16^ A diagnosis rewards the dysfunctional posture with an unsuitable label, providing credibility to the misconception of the sick patient. The diagnosis of malingering may be objective when the patient is observed to have the ability to perform an activity previously denied, exhibits lack of disuse atrophy, has symmetrical imaging studies, has a normal electrodiagnostic examination,^24^ and there is secondary gain.^25^ The subjects in these cases claimed impairments that were not explained by the objective examination, testing, and surveillance.

Malingering is of clinical and social concern. It takes away valuable medical resources from people who genuinely need attention. Both deception and fear of deception have consequences. Patients can get too much medical care when the doctor is deceived into thinking there is a real problem. Conversely, there is a risk of inappropriate medical care when the doctor fears deception. These consequences affect both the individual patient and the society.^26^ The diagnosis of malingering should be based on thorough history, physical examination, electrodiagnostic studies, imaging studies, and evaluation of all medical records.
